# Acoustic and Elastic Properties of a Blood Clot during Microbubble-Enhanced Sonothrombolysis: Hardening of the Clot with Inertial Cavitation

**DOI:** 10.3390/pharmaceutics13101566

**Published:** 2021-09-26

**Authors:** Laurent Auboire, Damien Fouan, Jean-Marc Grégoire, Fréderic Ossant, Camille Plag, Jean-Michel Escoffre, Ayache Bouakaz

**Affiliations:** Imaging and Brain Unit (iBrain, Inserm Unit 1253), Université de Tours, 37000 Tours, France; damien.fouan@gmail.com (D.F.); jean-marc.gregoire@univ-tours.fr (J.-M.G.); frederic.ossant@univ-tours.fr (F.O.); camille.plag@gmail.com (C.P.); jean-michel.escoffre@univ-tours.fr (J.-M.E.)

**Keywords:** microbubbles, stroke, ultrasound, rtPA, elastography, ultrafast imaging

## Abstract

Stroke is the second leading cause of death worldwide. Existing therapies present limitations, and other therapeutic alternatives are sought, such as sonothrombolysis with microbubbles (STL). The aim of this study was to evaluate the change induced by STL with or without recombinant tissue-type plasminogen activator (rtPA) on the acoustic and elastic properties of the blood clot by measuring its sound speed (SoS) and shear wave speed (SWS) with high frequency ultrasound and ultrafast imaging, respectively. An in-vitro setup was used and human blood clots were submitted to a combination of microbubbles and rtPA. The results demonstrate that STL induces a raise of SoS in the blood clot, specifically when combined with rtPA (*p* < 0.05). Moreover, the combination of rtPA and STL induces a hardening of the clot in comparison to rtPA alone (*p* < 0.05). This is the first assessment of acoustoelastic properties of blood clots during STL. The combination of rtPA and STL induce SoS and hardening of the clot, which is known to impair the penetration of thrombolytic drugs and their efficacy.

## 1. Introduction

Stroke is the second leading cause of death worldwide, with 6.7 million deaths in 2012 [1]. Each time a blood clot interrupts the flow circulation, this leads to ischemia, cell death, and inflammatory response. After the blood flow interruption, 1.9 million neurons are lost each minute and the ischemic brain ages 3.6 years each hour in absence of reperfusion [2]. Thrombolytic treatments have been developed to re-establish the blood flow as fast as possible. However, since 1995, the recombinant tissue type plasminogen activator (also known as rtPA) is the only clinically approved treatment to achieve this goal [3]. RtPA is an enzyme that cleaves the plasminogen present in the plasma and the clot into plasmin. Plasmin will break the clot fibrin network. Since 2015, the mechanical retrieval of blood clots, also named thrombectomy, has shown beneficial effects in patients suffering from ischemic stroke [4,5]. Both therapies have limitations, namely hemorrhages, a narrow time window of 4.5 h for rtPA [6], and the need to access a comprehensive stroke center for thrombectomy.

For these reasons, other therapies have been investigated. Sonothrombolysis (STL) relies on the i.v. injection of microbubbles (MB) subsequently exposed to an ultrasound to enhance the dissolution of the clot. Tachibana et al., in 1995, reported the first demonstration of the therapeutic potential of STL in the presence of a thrombolytic drug (urokinase) [7]. However, despite in-vitro and in-vivo investigations, as well as clinical trials, there is no clear evidence of better efficacy/safety ratio for STL compared to clinically approved treatment [8]. The current state of our knowledge shows the need to better understand the mechanisms involved in the STL process if we were to develop novel therapeutic approaches for clot dissolution.

Ultrasound have already been used to study the coagulation process without damaging blood samples. Thus, previous investigations reported that coagulation is a multi-step process by measuring the sound speed (SoS) and the backscattered echo from the clot with high frequency ultrasound [9], as well as by evaluating the elastic properties of blood during clotting [10]. Considering these previous results, the present study aims to explore the acoustic and elastic properties of the blood clot by measuring the SoS with a high frequency ultrasound and the Young modulus using supersonic shear wave imaging, during STL, with and without rtPA-based treatment.

## 2. Materials and Methods

All methods were carried out in accordance with relevant French guidelines and regulations.

### 2.1. Preparation of Human Blood Clots

As previously described [11], human blood samples were collected from healthy volunteers using an approved clinical research protocol. Informed consent of volunteers was obtained (French Blood Service, Tours, France). Blood was anti-coagulated with 3.2% sodium citrate (Becton, Dickinson and Company, Le Pont-de-Claix, France). Clotting was initiated by the addition of 500 mM CaCl_2_ (co-factor in the coagulation process) with specific parameters/features according to the exploration method:

(i) High frequency ultrasound imaging: high frequency imaging was carried out in order to assess the SoS of the clot before and after STL. A blood volume of 80 μL was placed in a custom polyolefin carrier (1.2 mm length, 1.6 mm inside diameter, 1.85 mm outside diameter). Polyolefin is highly biocompatible; thus, does not affect the protein activities [12]. In addition, polyolefin shows low ultrasound attenuation, which allows high frequency ultrasound exploration of the blood clot.

(ii) Supersonic shear wave imaging: as the spatial resolution of the Aixplorer^®^ ultrasound system (SuperSonic Imagine, Aix-en-Provence, France) to differentiate the elastic properties is in the order of 1 mm, larger blood clots were needed [13]. A custom holder was designed and a blood volume of 2.75 mL (6 mm thickness, 7 mm width, 60 mm length) was placed directly in the carrier.

The blood was allowed to clot (i.e., complete coagulation and fibrin network retraction) at 37 °C for 3 h (Incucell, MMM Medcenter Einrichtungen GmbH, München, Germany). Both methods preserved a physiological coagulation process and allowed performing serial tests without handling the blood clot.

### 2.2. Ultrasound Thrombolysis Setup

For STL, ultrasound waves were generated using a single-element and a lab-made ultrasound transducer with a center frequency of 1 MHz. Ultrasound waves and frequency were homogenous and constant. The transducer had a diameter of 15 mm and was naturally focused at 30 mm. It was driven by an electrical signal generated by an arbitrary waveform generator (Agilent, Santa Clara, CA, USA), then amplified by a power amplifier (ADECE, Veigné, France). As previously described [14], the peak negative pressure of the acoustic wave was measured in a separate setup using a calibrated PVDF needle hydrophone (diameter 0.2 mm; Precision Acoustics Ltd., Dorchester, UK) at the natural focal distance of the transducer.

### 2.3. In-Vitro rtPA Delivery

RtPA (Activase^®^, Genentech, San Francisco, CA, USA) was reconstituted and frozen after being split into 100 µL aliquots. RtPA was thawed prior to each experiment and diluted with recalcified human plasma to obtain a final concentration of 3 µg/mL (i.e., the human steady state concentration in stroke thrombolysis) [15]. The flow system simulated the hemodynamic conditions of the middle cerebral artery (Table 1). This system was an open circuit consisting in a flexible silicone tubing. Both ends were connected to a reservoir filled with recalcified human plasma (French Blood Service, Tours, France). A programmable hotplate magnetic stirrer (RH basic 2, IKA^®^ Werke GmbH & Co. KG, Staufen, Germany) maintained the plasma temperature at 37 °C. A peristaltic pump (MCP Process IP65, Cole-Parmer GmbH, Wertheim, Germany) provided constant flow to the system.

A total of 16 blood clots per setup were divided into four experimental groups: (1) control group (i.e., *w*/*o* pharmacological treatment); (2) 3 µg/mL rtPA (i.e., the human steady state concentration in stroke thrombolysis); (3) STL group (i.e., injection of BR14^®^ microbubbles, subsequently ultrasound application); and (4) RtPA + STL (i.e., co-injection of rtPA and BR14^®^ microbubbles, subsequently ultrasound application). For the 3rd and 4th groups, a microbubble solution was injected at 10^6^ microbubbles/min.

### 2.4. Sonothrombolysis

STL consisted of exposing the blood clots to 1 MHz sinusoidal ultrasound waves with a pulse repetition frequency of 0.8 Hz, 100,000 cycles per pulse for 30 min in the SoS setup, and 60 min in the SWS setup; respectively. STL application was turned off during SoS and SWS measurements. The applied acoustic pressure (i.e., peak negative pressure) was set to 600 kPa to trigger inertial cavitation in the presence of microbubbles. Inertial cavitation was defined as the occurrence of broadband emissions detected by a calibrated PVDF needle hydrophone. These parameters were selected considering our previous work [16] and the positive results obtained by other groups with relatively similar parameters [17].

### 2.5. High Frequency Ultrasound Imaging and SoS Measurement

**Figure 1:**
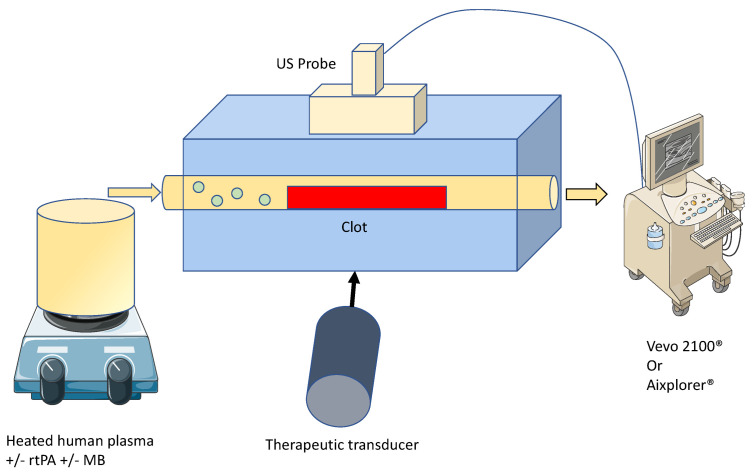
Schematic representation of the experimental setup 1 for high frequency imaging and SoS assessment (with Vevo2100^®^) and 2 for elasticity measurement (with Aixplorer^®^). The drawing of the ultrasound scanner and human plasma hotplate were obtained from Servier Medical Art of Servier under the Creative Common Attribution 3.0 France license.

**Figure 2:**
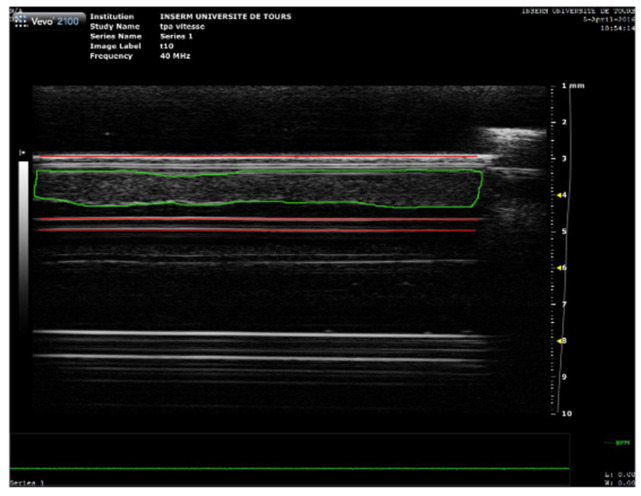
High frequency ultrasound imaging of blood clot. A 2D image of the blood clot obtained with the Vevo 2100^®^. The red lines delineate the anterior and the posterior walls of the tube, while the green shape delineates the clot.

A Vevo^®^ 2100 ultrasound scanner (VisualSonics Inc., Toronto, ON, Canada) with an MS-550D probe (22–55 MHz; 40 µm axial and 90 µm lateral resolutions) was used to acquire blood clot images every 1 min for 30 min (Figure 1). After acquisition, the images were analyzed using a custom MATLAB^®^ code to calculate the SoS, i.e., longitudinal wave, inside the blood clot. As depicted in Figure 2, the front and bottom of the tube were semi automatically delineated as well as the thickness of the clot in echographic images acquired with the Vevo^®^ 2100 ultrasound scanner. The distance between the 2 walls was first measured when water filled the tube. The distance was then measured when plasma filled the tube to estimate the SoS in plasma. Then, this distance was measured with the clot during the experiment every minute. The SoS in the clot was deduced by reporting the difference of SoS with and without the clot to the thickness of the clot.

### 2.6. Shear Wave Elastography Imaging

**Figure 3:**
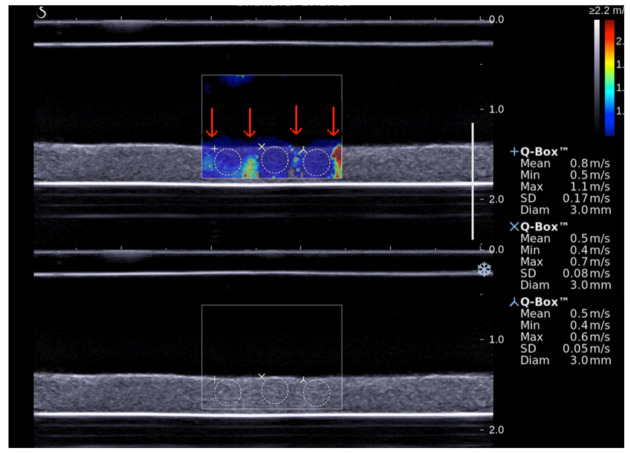
Shear wave ultrasound imaging of blood clot. Red arrows show ultrasound beam push artifacts. SWS was measured with Q-box^®^ software.

Aixplorer^®^ ultrasound scanner with an L15-4 probe (4–15 MHz, 1 mm axial resolution) [13,18] was employed to acquire blood clot elastographic images every 10 min for 60 min. After acquisition, elastic properties of the blood clot were quantitatively analyzed using the Aixplorer^®^ software. Shear wave speed (*SWS*) was measured for each region of interest (ROI) defined in the area exposed to the STL while avoiding ultrasound beam push artifacts (red arrows in Figure 3). Such artifacts were described by Lin et al. [19]. The Young modulus can be deduced from SWS for each ROI according the equation [20]:

$$SWS=\sqrt{E/3\rho }$$

where *E* is the Young modulus and *ρ* the density of medium. One notices that *ρ* is equal to 1000 kg/m^3^ by default on the Aixplorer^®^ ultrasound system while the blood has a slightly different density of 1080 kg/m^3^ [18].

### 2.7. Statistical Analysis

Descriptive statistics were performed using XLSTAT^®^ software (Addinsoft, Paris, France). Statistical analysis of the results was carried out using the non-parametric Kruskal–Wallis test and Dunn’s multiple comparison post-test. Significance was defined as *p* < 0.05.

## 3. Results

### 3.1. Sound Speed Measurement during Sonothrombolysis

**Figure 4:**
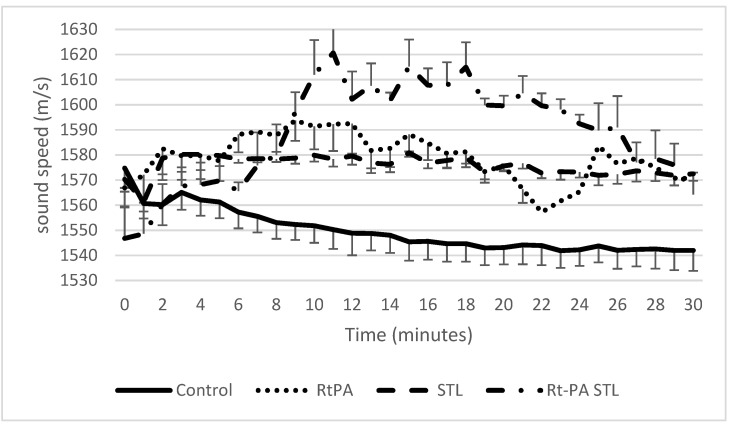
Measured SoS in the clot for the four different groups. Data expressed as mean ± standard error of the mean (SEM) were calculated from four independent experiments. Considering the control group, SoS decreases during the 30 min of the experiment, while it increases moderately in groups RtPA and STL. The rtPA-STL group shows the greatest increase of SoS at 10 min, and then shows a plateau before decreasing.

**Figure 5:**
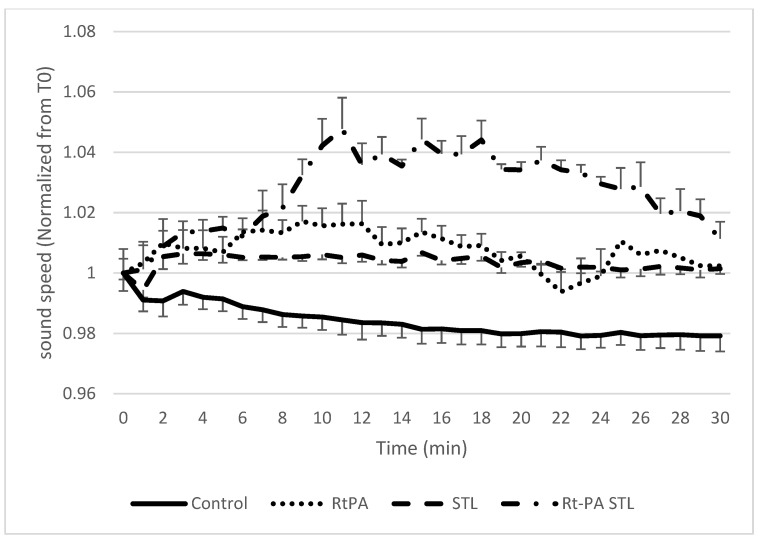
Measured SoS normalized to the initial sound speed in the clot for the four different groups. Data expressed as mean ± SEM were calculated from four independent experiments.

As previously described [11], blood clots were treated with either 3 μg/mL rtPA alone (i.e., the human steady state concentration in stroke thrombolysis), with STL alone (10^6^ BR14^®^ microbubbles/mL, and then ultrasound application), or with 3 μg/mL rtPA in combination with STL (i.e., co-injection of rtPA and BR14^®^ microbubbles, subsequently ultrasound application), and compared to the control condition (i.e., *w/o* pharmacological treatment). The SoS was then evaluated by measuring the distance between the anterior and the posterior walls of the tube. Indeed, the variation of this distance is proportional to the variation of the SoS. As shown in Figure 4, the SoS increased for all experimental groups compared to the control group during the total duration of the experiment. Figure 5 shows the same data normalized to the SWS at T0 to remove the inter variability among samples. A significant difference was found for rtPA, STL, and rtPA-STL groups compared to control group during the first 20 min (*p* < 0.05). In the rtPA group, three clots were detached from the wall of the tube after 20 min of experiments. One clot of the rtPA-STL group was detached from the wall before 30 min.

### 3.2. Assessment of SWS during Sonothrombolysis

**Figure 6:**
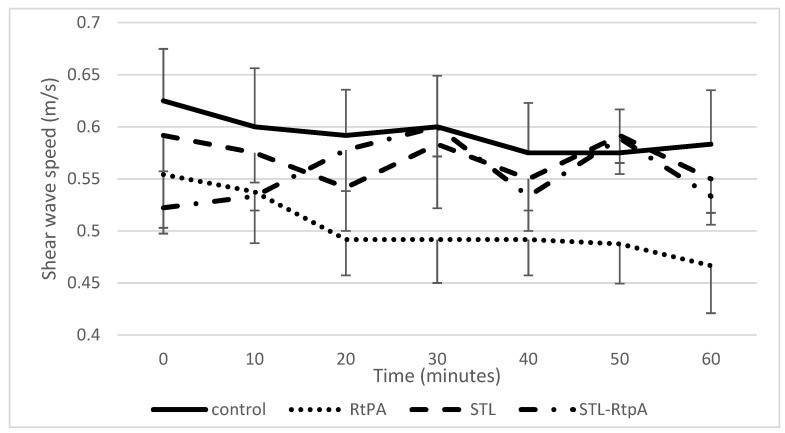
Measured SWS over the four different experimental conditions. Data expressed as mean ± SEM were calculated from four independent experiments.

The elastic properties of the blood clot were investigated using shear wave elastography imaging. For each experimental group, the SWS was measured every 10 min for 60 min and compared to the control group. Figure 6 showed that initial value of the SWS varies from 0.4 to 0.73 m/s. This variability can be explained because samples come from different donors. With control and rtPA conditions, the SWS decreased for 60 min, whereas it increased with rtPA-STL.

**Figure 7:**
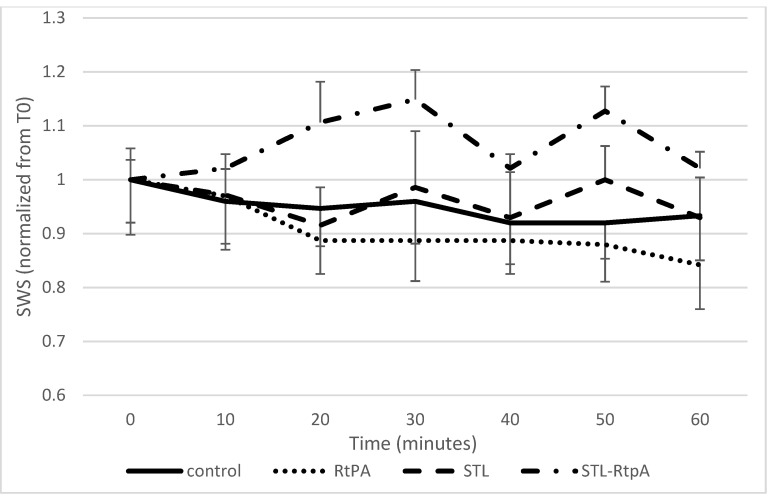
Measured SWS for the four experimental conditions, normalized to the initial value to better appreciate the effects of each condition independently from initial sample variability. This curve shows the evolution of SWS for 60 min. Data expressed as mean ± SEM were calculated from four independent experiments. Considering the rtPA group, SWS decreases during the 30 min of the experiment, while it decreases more moderately for groups RtPA and STL. The rtPA-STL group shows the greatest increase of SWS at 30 min.

Figure 7 showed the same data normalized to the SWS at T0 to remove the inter variability among samples. The addition of STL to rtPA led to a significant raise of the SWS of the clot compared to rtPA alone (*p* < 0.05). As described before, the variation of the SWS was proportional to the Young modulus. Interestingly, the action of the rtPA led to a diminution of the Young modulus, but when associated to STL, the Young modulus increased significantly (*p* < 0.05).

## 4. Discussion

The present study reports for the first time the effects of STL on acoustoelastic properties of blood clots. Indeed, our data showed that rtPA treatment alone or combined with STL induced a significant increase in SoS compared to the control group (Figure 5, *p* < 0.05).

In agreement with published data [21], our study reports that initial SWS values ranged from 0.4 to 0.73 m/s (Figure 6). In addition, we demonstrated that the clot elasticity decreased as a function of the exposure duration of the clot to rtPA (Figure 6 and Figure 7), a result in line with the experiment by Bernal et al. with urokinase (a drug similar to rtPA) [13]. Surprisingly, the blood clots exposed to rtPA-STL became stiffer than those treated with rtPA alone (Figure 6, *p* < 0.05). During the 8 to 30 min of experiment, the increase of SoS in the rtPA-STL group might have been due to the thinning of the clot, which exposed a greater part of the clot to the treatment. The diminution of SoS in the rtPA group observed between 20 and 24 min might have been related to a major structural change of theses clots just before they were destroyed, as three of four clots treated by rtPA detached from the wall after 20 min of experiment.

Although it is not possible to clearly identify the mechanism involved, some hypothesis can be discussed:

Firstly, these results might be explained by the mechanical activation of platelets: Jen et al. showed that clot rigidity was modulated by the contractile force generated by the platelet microfilament system [22]. Shigeta et al. proved that the use of microbubbles combined with ultrasound could induce platelets activation [23]. Tran et al. proved that the application of microbubbles and ultrasound is able to activate mechanical ionic channels in cells [24]. Considering these findings, we suggest that the STL-mediated mechanical stress can induce an activation of platelets leading to a clot contraction, which might clarify the raise of the SWS. In agreement with previous studies [25], the clot contraction might also be responsible of the significant increase in SoS. In addition, Samson et al. reported that tPA-mediated fibrinolysis facilitated clot contraction in-vivo, thus explaining the synergistic effect on SWS of STL combined with rtPA [26].

Secondly, these results might be associated to the biomechanical properties of fibrin network, which has a nonlinear elastic response to mechanical compression. Petit et al. demonstrated that STL lead to a compression of the fibrin network [17]. Kim et al. reported that a fibrin network became softer when exposed to a low compression force [27]. However, when this fibrin network is submitted to a high compression force (e.g., inertial cavitation), it becomes stiffer. Kim et al. explained this behavior by a complex interplay of entropic and enthalpic mechanisms accompanying structural changes, such as fibrin network densification and bending of fibrin fibers. Furthermore, Braaten et al. showed that a fibrin gel exposed and fixed in the presence of ultrasound exhibited an increase in density accompanied by a decrease in fiber diameter [28]. These results suggest that STL might induce a sufficient compression force on fibrin network to lead to its hardening.

Altogether, these data suggest that the modification of the SWS and the SoS is due to an active response of the clot to the STL induced mechanical stress. These results might influence the choice of ultrasound parameters in future STL research as the clot contraction and/or the fibrin network densification lower the ability of drugs to penetrate through the clot [29,30,31].

The main limitation of our study is related to the type of blood clot. Indeed, the blood clots used in the study were made with whole blood of healthy donors while previous studies reported that the cellular and molecular compositions of blood clots in ischemic stroke were different from those of healthy patients [32,33]. In addition, we considered that the density of the clot did not change during the experiments to interpret the variation of SWS. Nevertheless, Bernal et al. compared SWS values of clots obtained from classical rheology and with Aixplorer^®^, and found similar results, between 0.65 and 1 m/s, thus validating the use of the Aixplorer^®^ as a method to assess SWS in blood clots [21]. Lastly, our in-vitro device reproduced the hemodynamic conditions of the middle cerebral artery but not the blood pressure, which could modify the strain applied to the clot.

## 5. Conclusions

In summary and to the best of our knowledge, the present work reports, for the first time, the impact of STL on acoustic and elastic properties of clots. This study showed that rtPA, STL, and the combination of both led to an increase of SoS compared to the control group. However, rtPA combined with STL induced a larger increase of the SoS in the clot. SWS increased significantly when STL was added to rtPA. These results suggest that rtPA-STL under the inertial cavitation regimen might lead to a contraction of the clot and/or a densification of the fibrin network due to a mechanical stress.

Our findings provide new insight into the biophysical mechanisms involved in microbubble-assisted sonothrombolysis in ischemic stroke, and need to be confirmed by other approaches, such as the electrophysiological recording of platelets and optical exploration.

## Figures and Tables

**Table 1:** Parameters of flow systems.

	**High Frequency Ultrasound Imaging (SoS Setup)**	**Shear Wave Elastography Imaging (SWS Setup)**
Internal diameter of tube (mm)	1.6	3.2
Volume of recalcified human plasma (mL)	15	45
Constant flow (mL/min)	30	100
Duration of treatment (min)	30	60

## Data Availability

The data presented in this study are available on request from the corresponding author.
